# Improvement of phylogenetic method to analyze compositional heterogeneity

**DOI:** 10.1186/s12918-017-0453-x

**Published:** 2017-09-21

**Authors:** Zehua Zhang, Kecheng Guo, Gaofeng Pan, Jijun Tang, Fei Guo

**Affiliations:** 1 School of Computer Science and Technology, Tianjin University, 92 Weijin Road, Nankai District, Tianjin, People’s Republic of China; 2 Department of Computer Science and Engineering, University of South Carolina, Columbia, USA

**Keywords:** Phylogenetic analysis, Bayesian inference, Multi-chain Markov chain Monte Carlo, Conscious detection, Compositional heterogeneity

## Abstract

**Background:**

Phylogenetic analysis is a key way to understand current research in the biological processes and detect theory in evolution of natural selection. The evolutionary relationship between species is generally reflected in the form of phylogenetic trees. Many methods for constructing phylogenetic trees, are based on the optimization criteria. We extract the biological data via modeling features, and then compare these characteristics to study the biological evolution between species.

**Results:**

Here, we use maximum likelihood and Bayesian inference method to establish phylogenetic trees; multi-chain Markov chain Monte Carlo sampling method can be used to select optimal phylogenetic tree, resolving local optimum problem. The correlation model of phylogenetic analysis assumes that phylogenetic trees are built on homogeneous data, however there exists a large deviation in the presence of heterogeneous data. We use conscious detection to solve compositional heterogeneity. Our method is evaluated on two sets of experimental data, a group of bacterial 16S ribosomal RNA gene data, and a group of genetic data with five homologous species.

**Conclusions:**

Our method can obtain accurate phylogenetic trees on the homologous data, and also detect the compositional heterogeneity of experimental data. We provide an efficient method to enhance the accuracy of generated phylogenetic tree.

## Background

Phylogenetic analysis keeps an important role to understand current research in the biological processes and detect theory in evolution of natural selection. We extract the biological data via modeling features, and then compare these characteristics to study the biological evolution between species. The evolutionary relationship between species is generally reflected in the form of phylogenetic trees. Phylogenetic analysis can help to understand the evolutionary history of biological process, and become important data source for the development of large scale genomic data [1].

$$\begin{aligned} Q = \left(\begin{array}{cccc} -\mu(a\pi_{C} + b\pi_{G} + c\pi_{T}) & \mu a\pi_{C} & \mu b\pi_{G} & \mu c\pi_{T}\\ \mu g\pi_{A} & -\mu(g\pi_{A} + d\pi_{G} + e\pi_{T}) & \mu d\pi_{G} & \mu e\pi_{T}\\ \mu h\pi_{A} & \mu j\pi_{C} & -\mu(h\pi_{A} + j\pi_{C} + f\pi_{T}) & \mu f\pi_{T}\\ \mu i\pi_{A} & \mu k\pi_{C} & \mu l\pi_{G} & -\mu(i\pi_{A} + k\pi_{C} + l\pi_{G}) \end{array}\right) \end{aligned} $$

Many methods for constructing the phylogenetic tree, are based on optimization criteria, such as maximum parsimony, maximum likelihood and minimum evolution. Maximum parsimony (MP) approach [2, 3] examines all possible topologies or a certain number of topologies, which are likely to choose real phylogenetic tree or approximate phylogenetic tree with fewest evolutionary changes. Maximum likelihood (ML) approach [4, 5] tries to estimate trees by formulating a probabilistic model of evolution and applying known statistical method. It involves that phylogenetic tree yields the highest probability of evolutionary relationship. Minimum evolution (ME) approach [6] searches for the phylogenetic tree that minimizes total branch lengths. It is based on the assumption that the phylogenetic tree with smallest branch lengths is most likely to be the true one.

The correlation model of phylogenetic analysis assumes that phylogenetic trees are built on homogeneous data [7–10]. However, there exists a large deviation in the presence of heterogeneous data. As early as twenty years ago, there is first computational method [11] to detect heterogeneity problem, which makes people to doubt the credibility of phylogenetic analysis. Later, Markov model [12] of DNA sequence is used in the system development. Jukes-Cantor model [13] has been improved and taken into account unequal nucleotide compositions, different rates of changes from one nucleotide to another, variations in the form of invariant sites, and discrete gamma-distributed rates of variable sites. At the same time, researchers realize that the process of evolution would be different because of various evolutionary trees. It is obvious that the global rate can be often observed in fast and slow evolutionary species.

In this paper, we use maximum likelihood and Bayesian inference method to establish phylogenetic trees; multi-chain Markov chain Monte Carlo sampling method can be used to select optimal phylogenetic tree, resolving local optimum problem. We use two different instantaneous rate matrices, which is symmetrical and implies time-reversibility. We allow more than one composition vector to model compositional heterogeneity, because the overall model is tree-heterogeneous. The analysis is not reversible, and the likelihood depends the position of root. Compared to bootstrapping, Markov chain Monte Carlo yields a much larger sample of trees in the same computational time.

The correlation model of phylogenetic analysis assumes that phylogenetic trees are built on homogeneous data, however there exists a large deviation in the presence of heterogeneous data. The sample of trees produced by Markov chain Monte Carlo is highly auto-correlated, whereas many fewer bootstrapping replicates are sufficient. We make a conscious detection of phylogenetic tree produced by multi-chain Markov chain Monte Carlo sampling, analyzing multiple sampling and comparing different samples obtained from estimated values. We use conscious detection to solve compositional heterogeneity. Our method is evaluated on two sets of experimental data, a group of bacterial 16S ribosomal RNA gene data, and a group of genetic data with five homologous species. Our method can obtain accurate phylogenetic tree on the homologous data, and also detect the compositional heterogeneity of experimental data. We provide an efficient method to enhance the accuracy of generated phylogenetic tree.

## Method

We construct a phylogenetic tree for a set of DNA sequences. Our method generally contains following processes: aligning sequence [14–16], building phylogenetic trees, and selecting phylogenetic tree.

### Aligning sequence

The genetic information storage location has some differences on distinct species, such as information length and carrier of genetic information. These differences will affect our subsequent analysis. Therefore, we should arrange all possible similar sites in the same position, via a progressive algorithm of multiple sequence alignment. We adopt representational evolutionary multiple sequence alignment algorithm, called ClustalW [17–19]. It displays the alignment score, in form of identities, similarities and differences, and a guide tree of evolutionary relationship between aligned sequences.

### Building phylogenetic trees

The phylogenetic tree consists of many nodes and branches, where the node represents a taxon, namely species or sequence; the branch represents the evolutionary relationship between species [20, 21]. All nodes are divided into external nodes and internal nodes. In general, the external node represents actual observed taxon, the internal node represents location of evolutionary event.

#### Phylogeny model

Given the genetic information, we need the specific phylogeny model to predict evolutionary tree. First, we use the substitution model in terms of conversion rate. In general, the instantaneous conversion matrix is expressed as follows.

where this matrix specifies the rate of change from nucleotide *i*-row to nucleotide *j*-column. The nucleotides are in the order *A*,*C*,*G*,*T*. The stationary frequencies of nucleotides (*π*_*A*_, *π*_*C*_, *π*_*G*_, *π*_*g*_) are obtained by letting the substitution process run for a very long time.

The instantaneous conversion rate matrix describes the ratio of substitutions in a short period of time, but we need to calculate probabilities of changes in a certain period of time. Then, the probability matrix can be calculated as follows.

$$ P(t) = e^{Qt} $$

where *Q* is the instantaneous rate matrix, *t* is the branch length.

For a variety of evolutionary trees, we can calculate the likelihood of each phylogenetic tree. We need to consider the transformation between one external node and one internal node, and also consider the transformation between two internal nodes. For a specific site, we can calculate the likelihood of phylogenetic tree as follows.

$$L = \sum_{y}\pi_{y_{2s-1}}\prod_{k=1}^{s}p_{y_{\sigma(k)},x_{k}}(v_{k})\prod_{k=s+1}^{2s-2}p_{y_{\sigma(k)},y_{k}}(v_{k})  $$

where *x* and *y* are the external node and the internal node, respectively. *σ*(*k*) is the prefix index of *k*, *v*_*k*_ is the branch length between *y*_*σ*(*k*)_ and *x*_*k*_/*y*_*k*_. External nodes are *s* input sequences, that is, *s* species; according to the graph theory, we can get a total of 2*s*−1 internal nodes.

#### Log-likelihood

We assume that all sites are independent with each other. We can calculate the likelihood of each site [22], and then multiply them together to get final likelihood of phylogenetic tree.

We put all possible permutations, and then calculate the likelihood of all possibilities. For a specific site, the likelihood is the sum possibility of all internal nodes, denoted by *L*_*j*_. The likelihood of all sites can be calculated as follows.

$$\ln L = \sum_{j=1}^{N}L_{j}  $$

where *N* refers to the length of sequence and the total number of sites.

#### Bayesian inference

We can use Bayesian inference [23] to produce the posterior probability of *i*-th phylogenetic tree, *τ*_*i*_, as follows.

$$f(\tau_{i}|X) = \frac{f(X|\tau_{i})f(\tau_{i})}{\sum_{j=1}^{B(s)}f(X|\tau_{j})f(\tau_{j})}  $$

where *f*(*τ*_*i*_|*X*) is the posterior probability of *τ*_*i*_, *f*(*X*|*τ*_*i*_) is the likelihood of *τ*_*i*_, and *f*(*τ*_*i*_) is the prior probability of *τ*_*i*_. *B*(*s*) is the number of all possible trees.

### Selecting phylogenetic tree

Typically, the posterior probability of phylogenies cannot be calculated analytically, but it can be approximated by sampling phylogenetic trees from the posterior probability distribution.

#### Markov chain Monte Carlo

Markov chain Monte Carlo (MCMC) [24] can be used to sample phylogenies according to their posterior probabilities. The Metropolis-Hastings-Green (MHG) algorithm is an MCMC method that has been used successfully to approximate posterior probabilities of trees. MHG algorithm constructs a Markov chain with the stationary frequency of posterior probability. The current state is denoted as *τ*, and a new state is proposed as $\tau ^{'}\phantom {\dot {i}\!}$. The new state is accepted with probability as follows.

$$\begin{aligned} R & = min\left(1, \frac{f(\tau^{'}|X)}{f(\tau|X)} \times \frac{f(\tau|\tau^{'})}{f(\tau^{'}|\tau)} \right) \\ & = min\left(1, \frac{f(X|\tau^{'})f(\tau^{'})/f(X)}{f(X|\tau)f(\tau)/f(X)} \times \frac{f(\tau|\tau^{'})}{f(\tau^{'}|\tau)}\right) \\ & = min\left(1, \frac{f(X|\tau^{'})}{f(X|\tau)} \times \frac{f(\tau^{'})}{f(\tau)} \times \frac{f(\tau|\tau^{'})}{f(\tau^{'}|\tau)}\right)  \end{aligned} $$

**Fig. 1:**
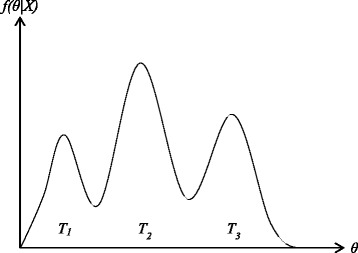
Markov Chain Monte Carlo method can only get the local optimal result in range *T*_1_ or *T*_3_

One important problem of MCMC method is that we can only get the local optimal result, but not the global optimum. As shown in Fig. 1, if the current state is at the peak of *T*_1_, because of the jump decision, the probability of next state must be less than one of current state, so MCMC method may get *T*_1_, but miss better *T*_2_.

#### Multi-chain Markov chain Monte Carlo

**Fig. 2:**
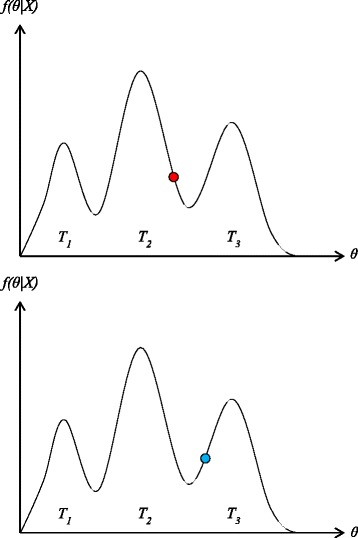
Cold Chain and Heat Chain for obtaining the global optimum: *red point* is used for heat status and *blue point* is used for cold status

When the distribution becomes flat, Multi-Chain Markov Chain Monte Carlo (MCMCMC) is easy to get down from the peak of local optimum, and then try to get more states. We set a cold chain, and rest of heat chains obtained by heat values. The heat value is obtained as follows.

$$ \beta_{i} = \frac{1}{1 + c(i-1)}  $$

where *c* is the heat coefficient according to the specific experimental data, *i* is the chain number. The state value of *i*-chain is calculated as $\phantom {\dot {i}\!}f_{i}(s) = f_{1}(s)^{\beta _{i}}$. Easy to see, the distribution is more gentle, as shown in Fig. 2.

Exchange occurs between two selected chains, and the exchange rate is determined as follows.

$$ R = \frac{f_{i}(s_{j})f_{j}(s_{i})}{f_{i}(s_{i})f_{j}(s_{j})}  $$

where *s* is the state of chain, *f*(*s*) is the state *s* corresponding to the state value in the special chain. When *R* is more than or equal to 1, it must be exchanged; when *R* is less than 1, it may be exchanged with probability value.

#### Conscious detection

The correlation model of phylogenetic analysis [9] assumes that phylogenetic tree is built on homogeneous data, therefore there exists a large deviation in the presence of heterogeneous data. We use conscious detection to solve compositional heterogeneity. We make a conscious detection of phylogenetic tree, analyze multiple sampling, and compare different samples obtained from estimated values. We extract the partial data from original data and form a new data set. Hundreds of data sets are used to generate different phylogenetic trees, and then get the support rate of different branches in the phylogenetic tree generated by actual data.

For *m*×*n* data set matrix, we select a random number from 1 to *n*, and obtain the column corresponding to this random number as re-sampling data for the first column; then repeat the above step to obtain re-sampling data of the second column, and so on. After *N*-loops selection, we get the final data set with same length of the original data set. For obtained data set, we analyze the phylogenetic tree according to phylogenetic analysis. Finally, we get *N* phylogenetic trees and their posterior probabilities, and analyze the genetic information.

## Results and discussion

Our method is evaluated on two sets of experimental data, a group of bacterial 16S ribosomal RNA gene data, and a group of genetic data with five homologous species.

### Experimental environment

We use Think Station S30 Workstation, and all programs are carried out on Ubuntu 14.04 64bit operating system, Intel Xeon E5-2620, 6 core 12 threads A-2 processor, 32G DDR3 1333MHz memory. We also use experiment softwares, such as multiple sequence alignment on CLUSTALX 2.0 [25, 26] and simulation test on JMODELTEST 2.17. The experimental data source is from National Center for Biotechnology Information (NCBI) database.

### Compositional heterogeneity in bacterial 16S genes

Our development system is applied to a problematic data set of bacterial 16S genes [27]: *Deinococcus*, *Thermus*, *Bacillus*, *Thermotoga*, and *Aquifex*. Specific information is shown in Table 1.

**Table 1:** Bacterial 16S genes: *Deinococcus*, *Thermus*, *Bacillus*, *Thermotoga*, and *Aquifex*

Organism	Accession	Type
*Thermus thermophilus*	NR_037066	complete sequence
*Bacillus subtilis*	NR_102783	complete sequence
*Thermotoga maritima*	NR_029163	complete sequence
*Aquifex pyrophilus*	NR_029172	partial sequence
*Deinococcus radiodurans*	NR_074411	complete sequence

**Fig. 3:**
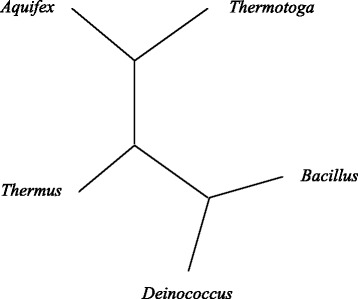
Predicted evolutionary tree on Bacterial 16S Genes

Our method produces the phylogenetic tree on 16S genes. We get prediction result with a tree (*Deinococcus*, (*Aquifex*, *Thermotoga*), (*Thermus*), *Bacillus*), as shown in Fig. 3. As we can see, *Thermotoga* and *Aquifex* are connected together, *Bacillus* and *Deinococcus* are connected together.

**Fig. 4:**
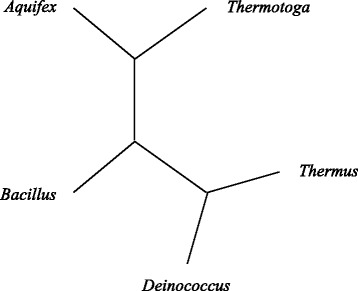
Actual evolutionary tree on Bacterial 16S Genes

However, other biological evidence, according to their actual evolutionary relationship, should introduce actual phylogenetic tree ((*Aquifex*, *Thermotoga*), (*Deinococcus*, *Thermus*), *Bacillus*), as shown in Fig. 4.

#### Conscious detection

Here, we re-sample 100 groups of data set, and construct one phylogenetic tree for each group of data set. Experiment results on 67 groups of data set are the same with their actual evolutionary relationship, as shown in Table 2. Based on conscious detection, we can correct the experimental data, in order to get the actual phylogenetic tree.

**Table 2:** Experiment results of our method with conscious detection on bacterial 16S genes

Posterior probability	Experimental groups
100%	17
[95*%*,100*%*)	28
[80*%*,95*%*)	12
[50*%*,80*%*)	10

### Homologous experiment

We adopt homologous gene sequences to construct the evolutionary tree, and find out evolutionary relationship. We use five species of albumin and c-myc mRNA genes [28]: *fish*(*Actinoptergyii*, *Salmo salar*), *frogs*(*Amphibia*, *Xenopus laevis*), *birds*(*Aves*, *Gallus gallus*), *rodents*(*Rodentia*, *Rattus norvegicus*) and *humans*(*Primates*, *Homo sapiens*), as listed in Table 3.

**Table 3:** Homologous data of albumin genes and c-myc mRNA genes

Species	Organism	Accession (albumin)	Accession
			(c-myc mRNA)
*Actinoptergyii*	*Salmo salar*	X52397	M13048
*Amphibia*	*Xenopus laevis*	M18350	M14455
*Aves*	*Gallus gallus*	X60688	M20006
*Rodentia*	*Rattus norvegicus*	J00698	Y00396
*Primates*	*Homo sapiens*	L00132	V00568

**Fig. 5:**
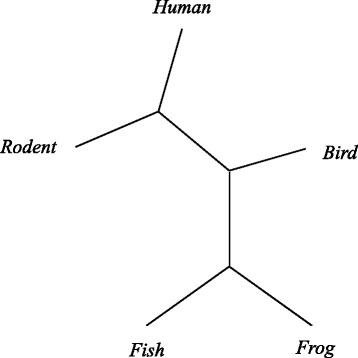
Evolutionary tree on albumin and c-myc mRNA genes

Our method produces similar experiment results on albumin and c-myc mRNA genes. We get result with a tree (*frog*, (*human*, *rodent*), (*bird*), *fish*), as shown in Fig. 5. As we can see, human and rodent are connected together, frog and fish are connected together. Experiment results on albumin and c-myc mRNA genes are the same with their actual evolutionary relationship.

### Xanthine dehydrogenase from drosophila

**Fig. 6:**
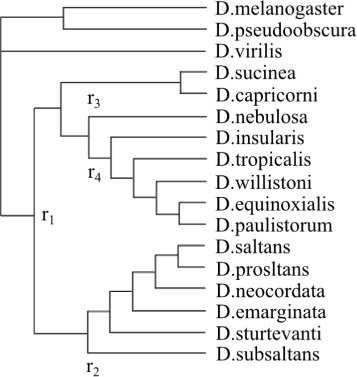
Rooting Drosophila saltans and willistoni groups

We analyze the root of Drosophila saltans and Drosophila willistoni groups, as outgroup rooting with the Xdh gene [29]. Based on morphology, we got the most credible root as shown in the root position *r*_1_ in Fig. 6, as well as based on deletion of an intron in the willistoni group-specific Adh gene. The outgroup is D. virilis, D. pseudoobscura and D. melanogaster. When only the ingroup is used, an acceptable phylogeny can be generated, which is consistent with the known relationships derived from morphological characters. When outgroup taxa are used in the analysis, depending on different model or method, the ingroup’s root position became unstable. This situation is resulted by the compositional differences, especially the ones between ingroup and outgroup taxa.

Four different roots indicated by positions *r*_1_- *r*_4_ in Fig. 6, the points where the outgroup attach to the ingroup on, are found by various methods. Here, the entire analysis’s overall root and the outgroup root position can be distinguished from each other, numbered as in Fig. 6. When accommodating the heterogeneous composition, this model can recover the outgroup root position *r*_1_. A distance-based analysis can overcome compositional heterogeneity, finding the preferred root position *r*_1_. We produce on these data to choose a model using the tree rooted at position *r*_1_, with the expectation that our choice of model is independent on other roots. A search for the GTR+SS model using PAUP finds a tree rooted at position *r*_2_. A Bayesian analysis using MrBayes also finds a tree rooted at position *r*_2_.

## Conclusions

In our paper, maximum likelihood, Bayesian inference method and multi-chain Markov chain Monte Carlo sampling are used to build and select global optimal phylogenetic tree. And also, compositional heterogeneity problem is solved by using conscious detection. When evaluated on two sets of experimental data, our method is efficient and accurate to generate phylogenetic tree and detect the compositional heterogeneity.

## Abbreviations

DNA: Deoxyribonucleic acid
RNA: Ribonucleic acid
mRNA: Messenger RNA
MP: Maximum parsimony
ML: Maximum likelihood
ME: Minimum evolution
MCMCMC: Multi-Chain Markov Chain Monte Carlo
MCMC: Markov chain Monte Carlo
MHG: Metropolis-Hastings-Green
